# Microglial activation in spaceflight and microgravity: potential risk of cognitive dysfunction and poor neural health

**DOI:** 10.3389/fncel.2024.1296205

**Published:** 2024-02-15

**Authors:** Zihan Li, Jiarui Wu, Tianyuan Zhao, Yiyun Wei, Yajing Xu, Zongjian Liu, Xiaoqiong Li, Xuechai Chen

**Affiliations:** ^1^Beijing International Science and Technology Cooperation Base for Antiviral Drugs, College of Chemistry and Life Science, Beijing University of Technology, Beijing, China; ^2^Department of Rehabilitation, Beijing Rehabilitation Hospital, Capital Medical University, Beijing, China; ^3^School of Life Sciences, Beijing Institute of Technology, Beijing, China

**Keywords:** microgravity, microglia, CNS, neuroinflammation, synaptic plasticity

## Abstract

Due to the increased crewed spaceflights in recent years, it is vital to understand how the space environment affects human health. A lack of gravitational force is known to risk multiple physiological functions of astronauts, particularly damage to the central nervous system (CNS). As innate immune cells of the CNS, microglia can transition from a quiescent state to a pathological state, releasing pro-inflammatory cytokines that contribute to neuroinflammation. There are reports indicating that microglia can be activated by simulating microgravity or exposure to galactic cosmic rays (GCR). Consequently, microglia may play a role in the development of neuroinflammation during spaceflight. Prolonged spaceflight sessions raise concerns about the chronic activation of microglia, which could give rise to various neurological disorders, posing concealed risks to the neural health of astronauts. This review summarizes the risks associated with neural health owing to microglial activation and explores the stressors that trigger microglial activation in the space environment. These stressors include GCR, microgravity, and exposure to isolation and stress. Of particular focus is the activation of microglia under microgravity conditions, along with the proposal of a potential mechanism.

## 1 Introduction

For nearly five decades, numerous space stations have been established in the near-Earth orbit zone in various nations. These stations serve as outposts for deep space exploration missions. Space presents a unique environment with reduced gravity and various environmental, operational, and psychological stressors. Notably, the challenges of microgravity and ionizing radiation in long-term space missions are significant for astronauts. Numerous experiments have been conducted to simulate microgravity and space radiation to understand the damage caused by extended spaceflight. These factors can impact astronaut health and cognition, potentially disrupting mission execution. Many astronauts stationed on the International Space Station (ISS) for extended periods have reported immune system issues (Crucian et al., 2016). Blood analysis during spaceflight has revealed abnormal cytokine activation in the peripheral immune system, suggesting the potential for excessive inflammatory responses (Crucian et al., 2013; Chang et al., 2015; Buchheim et al., 2019; Krieger et al., 2021).

Microglia, the innate immune cells of the central nervous system (CNS), play a crucial role in defending the brain and spinal cord. It is widely recognized that microglia originate from macrophages in the yolk sac, emerging around embryonic day 7.25 (E7.25) (Ginhoux et al., 2010). Microglia in the CNS are isolated from other immune cells and maintain homeostasis through self-renewal. Their essential functions include monitoring changes in the microenvironment, conducting physiological housekeeping, and protecting against harmful agents (Colonna and Butovsky, 2017; Borst et al., 2021). Several clinical and neuropathological studies have demonstrated that microglial activation plays a pivotal role in neuroinflammatory and neurodegenerative diseases. Microglia make up approximately 10–15% of all cells in the adult brain (Xavier et al., 2014). Generally quiescent, microglia can activate an amoeboid phenotype in response to various pathological stimuli in the CNS. They can adopt either a pro-inflammatory (M1) or anti-inflammatory (M2) phenotype, known as classical and alternative activation, respectively (Guo et al., 2022). Current research suggests a dynamic balance between these phenotypes in the cerebral microenvironment. However, the M1/M2 concept has been challenged by recent observations of diverse microglial states and functions in development, aging, and disease. It suggests (Paolicelli et al., 2022) that microglial activation is a highly dynamic process. There is ongoing debate regarding the use of gene or protein markers to identify microglial states.

**FIGURE 1:**
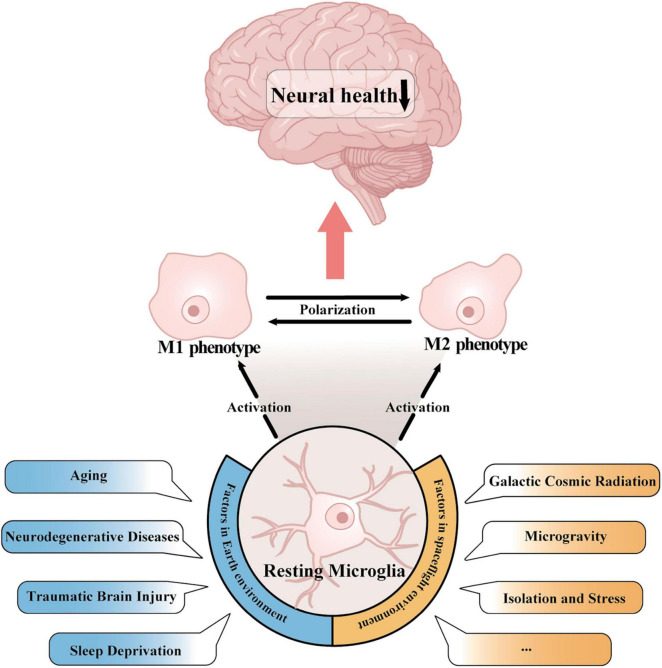
Activation of microglia in Earth’s and spaceflight environments. In both the Earth environment, which includes aging, neurodegenerative disease, traumatic brain injury, and sleep deprivation, and in the space environment, which includes cosmic radiation, microgravity, and stress exposure, microglia can switch from a resting state to a pro- or anti-inflammatory phenotype. Activated microglia regulate the transition between the two inflammatory phenotypes through polarization, which ultimately has an impact on human cognitive function and neural wellbeing.

Various pathological stimuli on Earth, such as aging (Shen et al., 2021; Goldman et al., 2022), neurodegenerative diseases (Brites and Vaz, 2014; Heneka et al., 2015), traumatic brain injury (TBI) (Kumar and Loane, 2012; Jassam et al., 2017), and prolonged sleep deprivation (Xue et al., 2019; Parhizkar et al., 2023), have been reported to induce microglial activation (Figure 1). Both the ability of microglia to monitor the brain and respond to insults diminishes with age (Hefendehl et al., 2014). Gulen et al. (2023) reported that in the aging brain, microglia show an active transcriptional phenotype associated with cognitive decline. A hallmark of aging is low-grade inflammation, and increased expression of melanoma 2 (AIM2) in the inflammasome regulates microglial activation via the complement pathway (Ye et al., 2023). In various neurodegenerative diseases, misfolded proteins like α-synuclein in Parkinson’s disease (PD) and amyloid-β (Aβ) in Alzheimer’s disease (AD) alter the phagocytic activity of microglia (Hansen et al., 2017; Wolf et al., 2017). Additionally, bioactive compounds such as astaxanthin (Kim et al., 2010, 2020), the sirt1 agonist resveratrol (Yang et al., 2017), hydroxytyrosol (Gallardo-Fernández et al., 2019; Zhang et al., 2020), and curcumin (Rahimifard et al., 2017) also induce microglial activation.

In spaceflight environments, galactic cosmic radiation (GCR), microgravity, space isolation and stress exposure are the major risks that have been reported to activate microglia (Figure 1). It was reported that single heavy ion radiation or multi-ion simulated galactic cosmic ray spectra (GCR sim) resulted in an inflammatory response and enhanced microglial activation, and this phenomenon can still be observed 12 months after irradiation (Parihar et al., 2016; Krukowski et al., 2018b). The impact of microgravity on the activation of microglia remains insufficiently defined, and experiments simulating microgravity on Earth have been employed for investigation. Hindlimb unloading (HU) represents a model of simulated microgravity tailored for rodents. *In vivo* assessments of microglial activity have been conducted utilizing HU, and subsequent to HU treatment, neuroinflammation and microglial activation have been detected in the hippocampus (Lin et al., 2020). Chelyshev et al. (2014) showed that simulated microgravity significantly increased the number of ionized calcium-binding adaptor molecule 1 (Iba1^+^) microglia in the central canal and dorsal root entry. Studies using BV-2 microglia, an immortalized cell line derived from mouse primary microglia, also revealed the activation of microglia in microgravity (Paulsen et al., 2015). These findings demonstrated that microgravity could induce the activation of microglia.

In this review, we first summarize the risks to neural health caused by microglial activation in normal earth environments, such as neuroinflammation, synaptic plasticity dysfunction, and abnormal phagocytosis. Therefore, we will discuss the stressors that can lead to microglial activation in the space environment, including GCR, microgravity, and stress exposure. Based on the known risk of microglial activation on Earth, we speculate that spaceflight may also cause nerve damage in the spaceflight environment induced by microglial activation. As microgravity is a major problem in spaceflight and can’t be avoid, we emphasize the activation of microglia in response to microgravity exposure and propose its potential mechanism and damage to the nervous system.

## 2 Microglial activation in the risk of cognitive function and neural health

**FIGURE 2:**
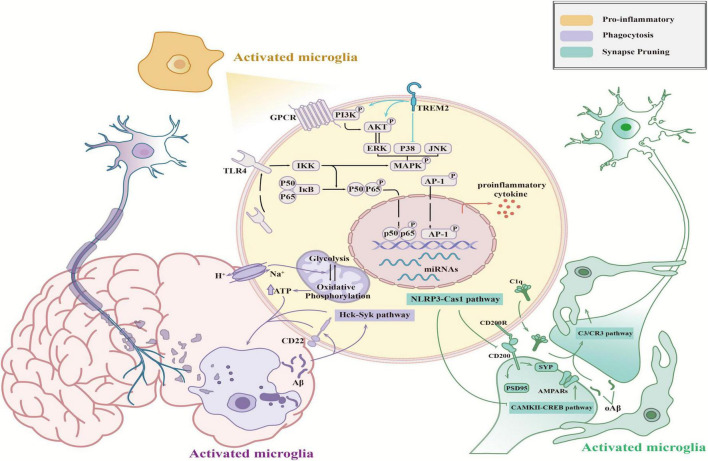
Functions of activated microglia in the CNS. Activated microglia mediate pro-inflammatory (orange), synaptic pruning (green), and phagocytosis (purple) effects, which influence neural health. The schematic shows the molecular mechanisms of these functions.

Microglia act as resident inflammatory cells in the CNS, releasing pro- and anti-inflammatory factors through activation to modulate the neuroinflammatory response. Furthermore, microglia also carry out their functions as immune cells via phagocytosis to participate in cellular debris removal and synaptic pruning (Bohlson and Tenner, 2023). However, neuroinflammation and phagocytosis play dual roles in neuro-homeostasis by both promoting neurological recovery and amplifying tissue damage, and their imbalance disrupts neuronal homeostasis and ultimately leads to cognitive decline and neurological-related diseases (Figure 2).

### 2.1 Neuroinflammation

Neuroinflammation impacts neural health through the production of neurotoxic mediators, which result from an exaggerated response to infection, aging, or surgical trauma. The build-up of these neurotoxic mediators interferes with repair mechanisms in microglia, affecting intracellular regulation. This leads to disruptions in axonal transport and mitochondrial function, ultimately resulting in neuronal death (de Araújo Boleti et al., 2020). Numerous studies have highlighted the dual pro-inflammatory and anti-inflammatory roles of microglia in neuroinflammation, emphasizing their significance in its development (Subhramanyam et al., 2019; Guo et al., 2022).

The nuclear factor-κB (NF-κB) pathway is a well-researched inflammatory pathway in microglia (He et al., 2023). Upon brain insults, microglia are quickly mobilized to recognize antigens. This triggers signal transduction that moves p65/p50 dimers from the cytoplasm to the nucleus, activating the NF-κB pathway (Sun et al., 2022). This activation leads to the secretion of pro-inflammatory cytokines, including interleukin (IL)-1, IL-6, and tumor necrosis factor α (TNF-α). Besides (Zhang et al., 2006) cytokine release, the NF-κB pathway also controls the production of reactive oxygen species (ROS), which compromise mitochondrial function and contribute to chronic neuroinflammation (Alvariño et al., 2021). Microglia induce excitotoxic neuronal death by overproducing inducible nitric oxide synthase (iNOS), a stress source that continuously activates microglia (Brown and Vilalta, 2015). Another key inflammatory pathway, the MAPK/AP-1 pathway, regulates the transcription factor activator protein-1 (AP-1) via activation of mitogen-activated protein kinase (MAPK) family members, including extracellular signal-regulated kinases 1 and 2 (ERK1/2), p38 MAPK, and c-Jun N-terminal kinase (JNK). The MAPK signaling pathway is well-established in microglial activation (Chen et al., 2022; Alhadlaq and Masocha, 2023; Aoki et al., 2023) and is known to stimulate the transcription of pro-inflammatory cytokines (Dhapola et al., 2021). It was reported that blocking the transcriptional activity of c-Jun could effectively ameliorate spatial learning impairment and memory deficits caused by microglial activation (Han et al., 2022; Shi et al., 2022; Xia et al., 2023). In addition, miRNAs (miR-155, miR-124, and miR-21) were found to modulate the microglial phenotype by regulating the expression of related proteins through the silencing of mRNA translation or degradation of transcripts and regulating the expression of pro-inflammatory signals and neuroinflammation development (Zhang et al., 2012; Su et al., 2016; Brás et al., 2020). Briefly, microglia are considered to be significant participants in neuroinflammation. Inhibiting the pro-inflammatory activation of microglia will become an important target for neuroinflammation therapy.

### 2.2 Dysfunction of synaptic plasticity

Microglia influence neuronal synapse formation and pruning through phagocytosis, playing a vital role in the wiring of neural circuits in the healthy brain. This function is crucial for learning, memory, behavior, and environmental adaptability (Miyanishi et al., 2021). The nucleotide-binding oligomerization domain-like receptor pyrin domain-containing 3 (NLRP3) inflammasome, a receptor protein on microglia membranes, was implicated in age-related cognitive decline in a mouse model. This involvement is through the NLRP3/Caspase-1 pathway, where NLRP3 expression negatively impacts the number or function of glutamate receptors and age-related cognitive functions of alpha-amino-3-hydroxy-5-methyl-4-isoxazolepropionic acid (AMPA) receptors, leading to synaptic plasticity and cognitive decline (Wang et al., 2022). The NLRP3/Caspase-1 pathway also attenuates hypoperfusion-induced dephosphorylation of calcium-calmodulin-dependent protein kinase II (CaMKII) and cognitive dysfunction (Wang et al., 2022). Li et al. (2009) noted that aging reduces the phosphorylation of CaMKII and its downstream molecule, cyclic adenosine monophosphate (cAMP) response element-binding protein. This reduction affects the expression of transcriptional genes like brain-derived neurotrophic factor, ultimately diminishing synaptic function and cognitive performance (Li et al., 2009). Certain receptors on microglia surfaces help maintain these cells in a quiescent state, contributing to CNS homeostasis. Overexpression of CD200 was shown to enhance the expression of two neuronal proteins, synaptophysin 1 and postsynaptic density protein 95 (PSD-95), regulating synaptic contacts in the hippocampus (Ojo et al., 2012).

It has been reported that complement-dependent phagocytosis assists microglia in synaptic elimination. During development, C1q and C3 localize to synapses and mediate synapse elimination via microglial phagocytosis, where they participate in the formation of mature neural circuits (Stevens et al., 2007). Microglia-mediated complement-dependent synaptic pruning precedes synaptic loss in the hippocampus of APP/PS1 AD transgenic mice, as indicated by an increase in C1q expression (Hong et al., 2016). In addition, soluble Aβ was also found to upregulate the expression of C1q, leading to cognitive decline. One possible explanation is that an increase in C1q promotes the activation of C3, which is located downstream of the classical complement cascade and ultimately mediates synaptic phagocytosis via C3/CR3 signaling in microglia (Hong et al., 2016). In conclusion, the formation and pruning of synapses participate in destroying the integrity of brain circuits, leading to impaired synaptic plasticity and cognitive impairment, eventually manifesting as synaptic plasticity dysfunction and cognitive impairment.

### 2.3 Abnormal phagocytosis

In addition to affecting the formation of synapses and brain circuits, phagocytosis of microglia also plays a crucial role in removing metabolic waste and misfolded proteins to maintain CNS homeostasis. Abnormal phagocytosis or impaired microglial ability directly affects cognitive function and neural health. For instance, the amount of myelin debris and protein aggregates increases during aging, and an imbalance in CNS homeostasis leads to a decrease in memory function, eventually leading to neurodegeneration (Hefendehl et al., 2014). Additionally, dysregulated microglia were demonstrated to lose the ability to clear Aβ at lesion sites, resulting in the aggregation of misfolded proteins and contributing to the pathogenesis of AD (Hansen et al., 2017).

The phagocytic function of microglia is influenced by various factors. CD22, a canonical B-cell receptor, was upregulated in aging microglia and involved in antiphagocytosis (Pluvinage et al., 2019). Lim et al. (2018) reported that the inhibition of Src family tyrosine kinases (SFKs)/hematopoietic cell kinase (Hck) or the knockout of the downstream factor SFK/Hck kinase spleen tyrosine kinase (Syk) resulted in significant attenuation of phagocytosis in microglia. Given that microglial phagocytosis requires high energy expenditure, the reprogramming and conversion of glycolysis and oxidative phosphorylation are important steps in this process (Song et al., 2022). It was reported that blocking the expression of Na^+^/H^+^ exchange isoform-1 (NHE1), which mediates H^+^ efflux in exchange for Na^+^ efflux, leads to a metabolic shift from glycolysis to oxidative phosphorylation and the generation of additional adenosine triphosphate (ATP). Changes in metabolism improve the phagocytic function of microglia and lead to increased phagocytosis (Song et al., 2018). Taken together, these findings suggest that a decrease in phagocytosis or dysregulation of metabolism could induce abnormal phagocytosis in microglia, ultimately influencing CNS homeostasis and exacerbating damage to neural health.

## 3 Microglial activation and cognitive dysfunction in spaceflight

In contrast to those of the Earth, contact with spaceflight conditions will bring a wide range of challenges from the external environment to lifestyle: GCR exposure, microgravity, isolation and stress exposure, circadian rhythm disruption, sleep disorders, and malnutrition (Gemignani et al., 2014; Petit et al., 2019; Baba et al., 2020; Kodaira et al., 2021; Desai et al., 2022). In this section, we focus on several major stressors that reportedly trigger neuroinflammation and microglial activation, potentially endangering cognitive function and neural health.

### 3.1 Galactic cosmic radiation

During spaceflight, astronauts are inevitably exposed to the space radiation environment, a complex field consisting of energetically charged particles (Nelson, 2016). Since spacecraft materials effectively shield electromagnetic waves, energetically charged particles originating from the Sun, GCR, solar particle events (SPEs), and Van Allen belts have become the major factors affecting the surface environment of the bodies of astronauts (Nelson, 2016). The GCR spectrum is composed of 90% protons, 9% helium (^4^He) ions, and 1% heavier ions and is regarded as the major source of radiation (Nelson, 2016; Cekanaviciute et al., 2018). Several ground-based simulation experiments have been conducted to reveal the damage to the CNS caused by GCR. It was demonstrated that microglia could be activated after exposure to different types of particles. Rola et al. (2008) reported that doses from 0.5 to 4 Gy of ^56^Fe irradiation had a negative effect on hippocampal neurons, which was associated with microglial activation and neuroinflammation. ^4^He radiation (5 and 30 cGy) was shown to lead to an increase in the number of activated microglia in the cortex, which induced neuroinflammation in the surrounding area and caused a continuous impact on neural health (Parihar et al., 2018). One concern is that rodents exposed to single heavy ions, such as ^48^Ti or ^16^O, exhibit a reduction in learning and memory knowledge over 12 weeks after radiation, which indicates that heavy-ion exposure may lead to long-term cognitive dysfunction (Parihar et al., 2016). Deficits in recognition memory were also observed in mice exposed to ^4^He or protons, revealing that all three components of the GCR could negatively affect cognitive function (Raber et al., 2016; Parihar et al., 2020). It is important to note that the selection of radiation doses in experiments is not standardized. In experiments to study the effects of the same particles, the radiation dose may range from 10 to 400 cGy, which can lead to insufficient comparability between different studies. Furthermore, the GCR spectrum is complex, and the many ions used for irradiation represent only a small fraction of the GCR spectrum that astronauts experience. Therefore, the doses used for single-particle radiation experiments can be much greater than the actual exposure dose in spaceflight (Rienecker et al., 2021).

Radiation-induced oxidative stress also emerges as a significant contributor to neurological harm and cognitive impairment, warranting careful consideration (Simpson and Oliver, 2020). ROS function as vital signaling molecules implicated in neuroinflammation. The brain is particularly vulnerable to this disruption owing to a confluence of factors, including heightened ROS generation, inadequate antioxidant defenses, and constrained regenerative capabilities. The damage-associated molecular pattern (DAMP) signaling pathway assumes a pivotal role in microglial activation and the facilitation of ROS production. The DAMP signaling pathway induced by damage in acute injury and neuroinflammation can respond to high mobility group box 1 (HMGB1), mitochondrial transcription factor A (TFAM), and cytochrome C via pattern recognition receptors (PRRs), such as CR3 and Toll-like receptor 4 (TLR4). Exposure to ^4^He radiation significantly upregulated the expression of TLR4 and the pro-inflammatory marker HMGB1 (Parihar et al., 2020), suggesting that GCR exposure may trigger oxidative stress, thereby inducing the activation of microglia.

Microglial depletion is considered a possible mechanism of radiation-induced cognitive impairment. As mentioned above, microglia can participate in synaptic remodeling through complement-dependent phagocytosis. After treatment with PLX5622 (PLX), a colony-stimulating factor 1 receptor (CSF-1R) inhibitor that induces depletion of microglia, the expression levels of two complement pathway mediators, C5aR and CD11b (CR3A), significantly decreased after radiation exposure (Feng et al., 2021). In addition, the expression levels of three key phagocytic markers, LAMP-1, CD206, and CD45, were also downregulated in the PLX treatment group compared with the irradiation alone group (Krukowski et al., 2018a), suggesting that both the complement cascade and the prominent phagocytosis mediated by microglia are affected by prolonged irradiation. Krukowski et al. (2018a) demonstrated that the depletion of microglia using PLX for approximately 2 weeks after radiation could prevent cognitive decline and dendritic spine loss in the hippocampus; moreover, Acharya et al. (2016) reported that the depletion of microglia during radiation may provide similar protective effects.

### 3.2 Microgravity

Microgravity is a unique gravitational environment resulting from the balance of centrifugal force exerted by orbiting spacecraft and Earth’s gravitational pull. In spacecraft, due to crew activities and vibrations from equipment, microgravity levels are approximately 10^−4^ to 10^−5^ g, while in CubeSats, it is around 10^−6^ g (Ferranti et al., 2020; ElGindi et al., 2021). Microgravity adversely affects various tissues and organs, causing muscle atrophy (Ohira et al., 2022), bone loss (Smith et al., 2014), cardiovascular (Aubert et al., 2016), and cerebrovascular diseases (Blaber et al., 2013). A primary concern is a decline in cognitive abilities due to microgravity (Liao et al., 2012; De la Torre, 2014; Cassady et al., 2016), critical to astronaut mission success.

Microgravity environments are generated in two ways. One is real microgravity, including drop towers, parabolic flights, sounding rockets, and orbital experiments; the other is simulated microgravity, which replicates microgravity effects on physiological responses using external force or gravity sensing delay. Various microgravity simulators or analogs have been developed for experiments from the molecular level to living organisms. Both real and simulated microgravity experiments should consider differences in microgravity conditions and duration.

#### 3.2.1 Real microgravity

Microgravity environments comprise drop towers, sounding rockets, parabolic flights, and orbital experiments. Drop towers provide high-quality microgravity, as low as 10^−6^ g, for a brief duration of 2.2 to 9.5 s (European Space Agency, 2022). Scientific research is typically conducted within a vacuum chamber to eliminate the influence of drag and friction forces (European Space Agency, 2022). In the field of biology, drop towers have been employed for conducting experiments related to membrane physiology and gravitaxis (Anken and Hilbig, 2004). Sounding rockets, which are suborbital vehicles, transport payloads into the upper atmosphere, reaching altitudes ranging from 250 to 350 km. Once they attain the desired altitude, the rocket releases its payload, subjecting it to a 5–20-min free fall before descending by parachute. Sounding rockets generally provide microgravity levels at or below 10^−5^ g (Ferranti et al., 2020). However, due to limited payload space, experiments require intricate designs and entail relatively higher experimental costs. Additionally, during launch and re-entry into the atmosphere, the payload may be exposed to elevated levels of hypergravity, potentially affecting the samples adversely. Parabolic flights simulate short-term microgravity (approximately 20 s), alternating between microgravity (10^−2^ g) and hypergravity (2 g) during each parabola (Ferranti et al., 2020). This model is utilized to investigate acute phenomena. A complete parabolic flight experiment typically encompasses approximately 30 successive parabolas, affording researchers the opportunity to analyze experimental procedures and make real-time adjustments to related parameters (Ferranti et al., 2020). Parabolic flight experiments offer a notable advantage by enabling the simultaneous execution of multiple experiments during a single flight, effectively optimizing time and reducing costs. However, it is essential to acknowledge that parabolic flight entails a combination of microgravity and hypergravity, which necessitates consideration when interpreting the results. Paulsen et al. (2015) demonstrated that during the microgravity phases, BV-2 microglia undergo a rapid and reversible reduction in intercellular adhesion molecule 1 (ICAM-1) expression on the cell surface. In human subjects, Schneider et al. (2008) observed that the microgravity phases of parabolic flights induced significant alterations in frontal lobe activity, a region closely associated with emotional processing and performance modulation.

Orbital experiments, including those with CubeSats, satellites, and the ISS, are recognized as the most expensive yet reliable platforms for microgravity research, offering prolonged and stable conditions. These in-orbit microgravity experiments necessitate a detailed experimental plan, involving an extensive development cycle, and the equipment used must possess a high degree of automation. To this point, *in vitro* experiments on microglia in the ISS context have not been documented. However, several experiments at the individual level have been performed. Mhatre et al. (2022) observed that, over a period of 34 days, oxidative damage in Drosophila subjected to spaceflight microgravity conditions could lead to elevated ROS levels, triggering a cascade of events resulting in neuronal damage. Additionally, significant climbing defects were noted in the spaceflight microgravity group (Mhatre et al., 2022). Another study involving long-term microgravity exposure (33 days) on female C57BL/6 mice reported behavioral alterations. In this study, the behavior of mice aboard the ISS was meticulously recorded and analyzed. It was observed that within 7–10 days post-launch, younger mice (aged 16 weeks) started showing unique circling behaviors, which eventually developed into coordinated group activities (Ronca et al., 2019). This circling behavior is hypothesized to be either a form of stereotyped motor behavior or an abnormal repetitive behavior.

Moreover, several cases of genetic or proteomic analyses of spaceflight mice of different durations have also been reported. Mao et al. (2018) investigated spaceflight condition-induced changes in protein expression in the gray and white matter of female C57BL/6 mice after a 13-day spaceflight mission. Enolase 2 (ENO_2_) was found to be upregulated in the gray matter, indicating potential impacts on microglial glycolysis. Moreover, arginase 1 (Arg1) was significantly upregulated, suggesting that microglia may be alternatively activated and play an anti-inflammatory role in counteracting the effects of microgravity. However, Holley et al. (2022) reported dysregulation of genes supporting innate immune responses, microglial function, and ROS generation in male C57BL/6 mice following 35 days of spaceflight. Notably, the triggering receptor expressed on myeloid cells 2 (Trem2), an inhibitor of neuroinflammation, was downregulated during spaceflight and the expression of apolipoprotein E (ApoE) increased, indicating the impairment of purinergic signaling which could lead to microglial activation (Victor et al., 2022). The duration of spaceflight may play a pivotal role in different responses to gene expression profiles and pathway regulation, as biological processes related to immune/inflammation were upregulated in another 91-day spaceflight experiment in which male C57BL/10 mice were used (Santucci et al., 2012).

A major threat to the success of space missions is insufficient performance and asthenia. Previous work has shown that various psychomotor functions, such as central postural functions, attention, limb position sense, and the central management of concurrent tasks, are impaired during spaceflight (Roll et al., 1993; Manzey and Lorenz, 1998). However, the role that microglia may play in these changes is still unknown. Obtaining microglial samples directly from astronauts is not feasible due to their unique distribution, and there are no current reports of *in vitro* cultivation of microglia in Spacelab. Future research on microglia in real microgravity conditions is needed.

#### 3.2.2 Simulated microgravity

Simulated microgravity replicates the physiological effects of microgravity by applying an external force or exploiting the delay in gravity perception, and the purpose of simulating microgravity analogs is to reproduce the effects of microgravity on organisms in specific aspects. Tools such as 1D/2D Clinostats, the Rotary Cell Culture System (RCCS), and 3D Clinostats/random positioning machines (RPMs) are recognized for their affordability and convenience in creating simulated microgravity environments on Earth. Clinostats achieve this simulation by continually altering the orientation of objects in relation to the gravitational vector, producing effects similar to actual microgravity, especially when these changes outpace the object’s gravitational response (Borst and Van Loon, 2009). Clinostats are categorized into different types based on rotation speed and the number of rotational axes. The RCCS, in particular, prefers faster rotation speeds to counteract sample sedimentation, making it more suitable for suspension cells. Additionally, it is crucial to address the shear forces generated by sample rotation when using an RPM. Modifying rotational parameters and preventing air bubble formation in the liquid medium are effective strategies to overcome these challenges (Borst and Van Loon, 2009; Wuest et al., 2017). By adjusting the rotation speed and the distance from the center, a simulated microgravity level of approximately 10^−4^ g can be achieved (Grimm et al., 2014). The operational convenience and affordability of Clinostats make them useful tools for preliminary exploration prior to conducting microgravity experiments, but the results obtained in simulated microgravity must be verified during real microgravity. Paulsen et al. (2015) demonstrated that BV-2 cells were activated and that ICAM-1 expression was downregulated under simulated microgravity conditions induced by a 2D clinostat at 60 rpm. Furthermore, a subsequent study showed that the downregulation of ICAM-1 was reversible in the microgravity phases of parabolic flight (Paulsen et al., 2015).

Rodent HU has been widely accepted by the scientific community as the rodent model of choice for simulating microgravity (No author list, 1992). In this model, the hindlimbs of rodents are raised to create a 30° head-down slope, which results in a cephalad fluid shift and prevents weight bearing on the hindquarters (Morey-Holton and Globus, 2002). It should be noted that as the mice are generally housed individually, the HU model may unintentionally lead to social isolation, potentially affecting their behavior (Tahimic et al., 2019).

In recent years, several studies have reported that microglia can be activated by HU treatment. Lin et al. (2020) demonstrated that following 7 days of HU treatment, the number of sites positive for the microglial marker Iba1 increased in the dentate gyrus, while the number of neural stem cells decreased. Minocycline inhibited the activation of microglia and reversed the damage. Similar results were also observed in the spinal cord. Prolonged simulated microgravity (30 days HU) increased the number of Iba1-positive microglia approximately 1.6-fold, and their morphology transformed into larger more arborized processes (Chelyshev et al., 2014). CD68, another indicator of microglial activation, was also significantly increased in the hippocampus, and the expression of three pro-inflammatory cytokines, IL-3, IL-12, and IL-17, was found to increase and be significantly correlated with CD68 upregulation (Rubinstein et al., 2022). In addition to the direct results of observing microglia, some studies have demonstrated that HU treatment induces oxidative stress injury in the brain (Mao et al., 2016; Rubinstein et al., 2022), which may induce the production of pro-inflammatory substances and create a self-perpetuating neurotoxic cycle (Rojo et al., 2014). Overall, the activation of microglia by microgravity exposure is associated with the upregulation of neuroinflammatory markers, oxidative stress damage, and the occurrence of neuroinflammation (Table 1). However, the mechanisms through which microglia respond to microgravity and are activated remain to be further investigated.

**TABLE 1:** Overview of the effects of ground microgravity simulators and analogs.

Platform	Subject	Duration	Observation
2D clinorotation	BV-2 cells	60 rpm for 24–96 h	BV-2 cells were found activated and decreased ICAM-1 expression (Paulsen et al., 2015)
HU	(Adult) male Sprague-Dawley rats	7 days	The number of Iba1-positive microglia increased in the dentate gyrus (Lin et al., 2020)
	(5–6 months) male C57BL/6J mice	14 days	Analysis of mRNA expression levels was performed and observed the upregulation of ion channel-related genes (Frigeri et al., 2008)
	(10 weeks) male Sprague-Dawley rats	21 days	Increased oxidative stress levels, pro-inflammatory cytokine levels, and permeability, damaged BBB ultrastructure (Yan et al., 2021)
	(6 months) female C57BL/6 mice	21 days	Increased oxidative stress injury in the brain (Bellone et al., 2016)
	(8 weeks) male Sprague-Dawley rats	28 days	Proteomics analysis was performed and increased glutamate receptor 1, glutamate receptor 4, and glutamic acid expression in hippocampus and decreased 5-hydroxytryptamine, dopamine, and γ-amino acid butyric acid (Wang et al., 2017)
	(16 weeks) female wild type C57BL/6NJ and MCAT transgenic mice	30 days	Increased CD68 expression and pro-inflammatory cytokines IL-3, IL-12, and IL-17 which were significantly correlated with CD68 upregulation (Rubinstein et al., 2022)
	Male C57B/6 mice	30 days	The number of Iba1-positive microglia approximately 1.6-fold and their morphology transformed into larger more arborized processes in the spinal cord (Chelyshev et al., 2014)

### 3.3 Isolation and stress exposure

The spatial isolation and social constraints inherent in spaceflight could produce a persistent and stressful environment for astronauts (Van Baarsen et al., 2009). Additionally, mission performance necessitates a high degree of mental acuity, which may further pressure and undermine mental stability. Although few comprehensive models can construct a spaceflight-isolation simulation system, in some special or extreme environments, long-term isolation can induce depression and negative adjustment reactions in crew members (Sandal et al., 1995; Sandal, 2000). Both clinical and preclinical studies have demonstrated that stress plays a significant role in the pathophysiology of depression, which consequently activates microglia (Kessler, 1997; Risch et al., 2009). Du Preez et al. (2021) demonstrated that chronic mild stress and social isolation could lead to several behavioral changes resembling depression and upregulate microglial activation in the dentate gyrus of the hippocampus in male mice. Furthermore, isolation-induced activation of microglia results in specific alterations in sex and brain regions (Vu et al., 2023). The volume, territory, and endpoints of microglia were shown to increase in the dorsomedial hypothalamus and hippocampal CA2 region in adult male mice following social isolation, while females showed an increase in only the hypothalamus (Vu et al., 2023). Current findings have demonstrated that social isolation and chronic stress may result in the activation of microglia, and these negative impacts could be sex-specific. Therefore, appropriate psychological and medication interventions are needed to improve the mental health of astronauts during missions.

## 4 Mechanistic assumptions of microglial activation in microgravity

**FIGURE 3:**
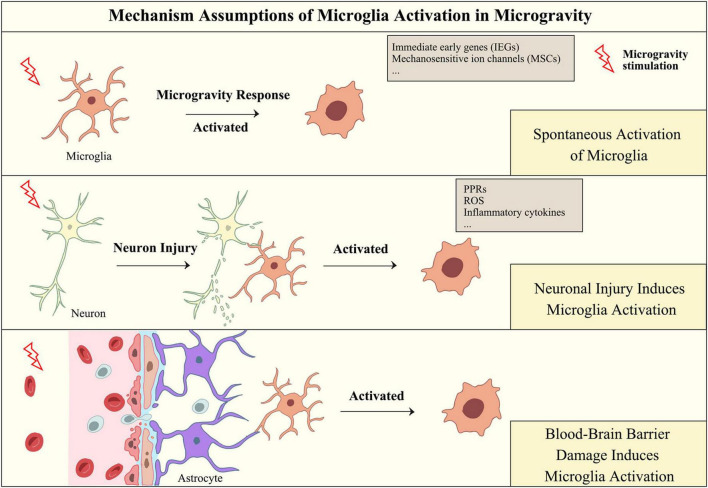
Hypothesis of the mechanism of microglial activation in microgravity. Microgravity may activate microglia through direct or indirect effects, including responding to microgravity signals through microglial receptors, responding to microgravity-induced damage to neurons and damaging the integrity of the blood-brain barrier.

Serving as innate immune cells in the CNS, microglia defend against insults and regulate the homeostasis of the brain microenvironment. In response to these stimuli, microglia can initiate a neuroinflammatory response, while persistent neuroinflammation, in turn, induces neurotoxicity, leading to neuronal damage. Although microglial activation in simulated microgravity has been reported, the specific mechanism of microgravity-induced microglial activation remains unclear. Considering the general pathways of microglial activation, it can be hypothesized that microglia may be spontaneously activated in microgravity conditions and may also be activated by neuronal injury and damage to the blood-brain barrier during spaceflight (as shown in Figure 3).

### 4.1 Spontaneous activation of microglia

The current limitation of microglial experiments in microgravity is that relatively few experimental reports on microgravity have been published, and most of them were conducted under simulated microgravity conditions. Nevertheless, some studies indicate potential avenues for considering spontaneous activation of microglia.

Immediate early genes (IEGs) are a type of gene that are activated rapidly in response to cellular stimuli. Several studies have reported that *c-fos* and *c-Jun* are regulated in simulated microgravity (Shi et al., 2021). Furthermore, ICAM-1, a transmembrane protein expressed on the surface of epithelial cells, endothelial cells, T cells, and macrophages, was also demonstrated to respond to microgravity stimulation (Buravkova et al., 2018; Lü et al., 2019; Nassef et al., 2019; Ratushnyy et al., 2019). ICAM-1 expression was observed to decrease quickly and reversibly on the surface of BV-2 cells following the microgravity phase in parabolic flight, but in long-term microgravity induced by orbital SIMBOX/Shenzhou-8, ICAM-1 expression increased in macrophage-like differentiated human U937 cells (Paulsen et al., 2015). The difference in the regulation of ICAM-1 between U937 and BV-2 cells under microgravity conditions may be attributed to differences in the source of the species or macrophage distribution (Paulsen et al., 2015). Additionally, opposite results were observed for the expression of ICAM-1 in primary human macrophages following different durations of spaceflight (Paulsen et al., 2015). These results demonstrated that ICAM-1 could be sensitive to the microgravity response in the monocyte/macrophage system and that changes in the expression of ICAM-1 may contribute to functional impairments in the innate immune system.

The regulation of ICAM-1 expression is a dynamic process that is closely linked to cytoskeletal function. Changes in the composition of the cytoskeleton were observed under both simulated and real microgravity conditions (Cogoli-Greuter, 2014; Thiel et al., 2019; Wu et al., 2022). A recent study showed that microglial reactivity requires the remodeling of microtubules (Rosito et al., 2023). Gitik et al. (2010) also demonstrated that the cytoskeleton is involved in the regulation of microglial phagocytosis via myosin light chain kinase (MLCK), Rho GTPases, and the effector Rho-kinase (ROCK). Interestingly, the gene expression of RhoA was upregulated in the mouse brain after a 35-day spaceflight mission (Holley et al., 2022). Although these transcriptomic results were based on the analysis of whole-brain tissue, we suggest that Rho GTPases may be involved in the activation of microglia in microgravity conditions. Another piece of supporting evidence for this hypothesis stems from the response mechanism of microgravity signals. Rho GTPases are recognized as key intracellular mechanotransduction mediators that can regulate multiple cell activities, and these regulatory effects are highly context dependent (Xie et al., 2023). Microgravity, especially cephalad fluid shifts and increasing organic pressure caused by microgravity, can be regarded as a type of mechanical stimulus. Microgravity-stimulated signals may trigger multiple mechanosensitive ion channels (MSCs) [e.g., Piezo1 (Chen et al., 2018) and transient receptor potential vanilloid 4 (TRPV4) (Adapala et al., 2016)] on the surface of microglia, thus initiating the intracellular transmission of microgravity-stimulated signals and ultimately inducing the activation of microglia. Previous studies have indicated that MSCs may act as important upstream agents of the microgravity response process in microglia (Wuest et al., 2018). For example, Piezo1 has been found to be essential for innate immunity. Solis et al. (2019) demonstrated that innate immune cells could initiate an inflammatory response via mechanically activated Piezo1 under cyclic hydrostatic pressure. The number of Iba1-positive cells in the hippocampus was increased after the injection of TRPV4 agonists, suggesting that TRPV4 stimulation may convert microglia to an activated phenotype (Wang et al., 2019). Additionally, increased levels of the neuroinflammatory marker NLRP3, apoptosis-associated spot protein, and cystatin-specific protease-1 were also observed, suggesting that neuroinflammation occurs after TRPV4 activation (Wang et al., 2019). While these studies do not directly reveal the mechanism of microglial activation in microgravity, they once again suggest that microgravity stimulation may be the major factor inducing microglial activation.

### 4.2 Neuronal injury induces microglial activation

Neurons are the main contributors to brain function. Given that the elimination of apoptotic neurons and the maintenance of microenvironmental homeostasis are functions of microglia, neuronal damage caused by microgravity exposure should be considered initiating events that trigger microglial activation. The ability to phagocytose apoptotic neurons enables microglia to rapidly converge toward the site of CNS damage after acute brain injury and transform into an ameboid form (Stence et al., 2001).

Microglial activation occurs in the early stages of neuroinflammation, during which the brain rapidly responds to the area of injury and persistence (Kawabori and Yenari, 2015). Simulated experiments with RPM have shown that microgravity is capable of damaging the formation and maturation of neural networks, leading to an increase in apoptosis (Pani et al., 2013, 2016). A study investigating the effects of microgravity on motoneurons revealed that the expression of caspase-3 was upregulated after 30 days of spaceflight, and fragmentation of neurons with formation structures similar to apoptotic bodies was observed in individual caspase 3-positive motoneurons (Porseva et al., 2017). Microglia can identify apoptotic neuronal fragments as antigens, potentially initiating their activation. Prolonged exposure to microgravity, in particular, could lead to enduring and chronic neuronal harm. While the phagocytic process of microglia concerning neuronal apoptosis is a matter of interest, there have been no published reports regarding the interaction between neurons and microglia in microgravity environments. Given the role of microglia in removing damaged cells, it is conceivable that neuronal apoptosis in microgravity conditions may prompt the activation of microglia.

### 4.3 Blood-brain barrier damage induces microglial activation

Microgravity-induced dysfunction of the blood-brain barrier (BBB) and neuroinflammation are believed to aggravate microglial activation. The integrity of the BBB relies on the coordinated signaling pathways facilitated through intercellular communication among various brain cells, including endothelial cells, pericytes, astrocytes, microglia, and oligodendrocytes (Segarra et al., 2021). Factors compromising BBB integrity include both cellular components, like endothelial cell dysfunction, and noncellular components, such as inflammatory mediators in the microenvironment (Takata et al., 2021). In cases of brain injuries, for instance, mild traumatic brain injuries, BBB disruption precedes the induction of neuroinflammatory responses through microglia and astrocyte activation (Wu et al., 2021). Chronic brain injury can result in prolonged BBB damage, sometimes lasting years (Hay et al., 2015), accompanied by the leakage of plasma proteins like fibrinogen, IgG, and albumin, which triggers microglial activation and cytokine release (Hooper et al., 2005). Recent findings from both simulated microgravity and actual spaceflight suggest that microgravity could impair BBB integrity. Yan et al. (2021) demonstrated that 21 days of HU treatment induced oxidative stress, damaged BBB ultrastructure, and reduced the expression of tight junction and adherens junction proteins in rat brains. Additionally, an increase in pro-inflammatory cytokines like TNF-α, IL-6, and interferon gamma (IFN-γ) was observed (Yan et al., 2021). A spaceflight study by Mao et al. (2020) provided pivotal evidence of downregulated zonula occludens-1 expression and increased aquaporin 4 in the hippocampus. This study also showed increased expression of perivascular reactive glial fibrillary acidic protein (GFAP) astrocytes in the hippocampus of spaceflight mice, suggesting astrocyte proliferation and hypertrophy in response to brain injury, contributing to BBB destruction through the release of chemokines and cytokines (Mao et al., 2020). Overall, existing findings indicate that the disruption of the BBB caused by microgravity could lead to the improper leakage of plasma proteins, dysfunction of brain endothelial cells, and gliosis, ultimately leading to the activation of microglia.

## 5 Prospects

Human space exploration is still in its nascent stages. Studies have identified potential risks to astronauts’ cognitive and neural health posed by spaceflights. One such risk factor is microgravity, a distinctive gravitational environment encountered during space missions. Prior research has reported microglia activation in response to simulated microgravity. Nevertheless, further research is necessary to elucidate the mechanisms through which microgravity activates microglia, particularly in actual spaceflight conditions (Santucci et al., 2012; Mao et al., 2018; Holley et al., 2022). Recent transcriptomic and proteomic analyses conducted during spaceflight, although based on whole-brain conditions without a specific intracellular focus, provide a direction for future research. In addition, another type of Earth-bound experimental model, centrifugation-induced hypergravity, was performed to study biological systems sensitive to changes in gravity. The effect of hypergravity could involve oxidative stress on the developing brain (Sajdel-Sulkowska et al., 2007), and the expression of IL-1β and TNF-α was also found to increase in the brains of rats repeatedly exposed to hypergravity (Liu et al., 2000). Although these findings do not directly demonstrate microglial activation under hypergravity conditions, they still indicate that changes in gravity may lead to inflammatory reactions and oxidative stress in the brain.

The utilization of animal models for spaceflight experiments is a widely used method to study the impact of microgravity. Nonetheless, variations in animal sex, age, and cage environment could result in differing findings. The possible risk of automated food disponder malfunction could also become an unstable factor, leading to the accidental death of subject animals. A spaceflight mission conducted on the unmanned Bion-M1 in 2013 resulted in a malfunction in the food dispenser, resulting in the survival of only approximately 50% of the mice (Andreev-Andrievskiy et al., 2014). The key point is that current animal experiments conducted in orbit have a common feature: the collection of tissue samples is arranged after the animals return to Earth. Therefore, animals experience significant hypergravity during the landing process, which interferes with the results. Processing samples in orbit after an experiment is a viable solution to avoid the influences of hypergravity, but the operator must undergo specialized training or participate in spaceflight missions with scientists, which increases the cost of space experiments. In addition, unlike other immune cells, such as macrophages found in the peripheral blood, the limited distribution of microglia within the mammalian brain poses difficulties in terms of isolation and subsequent study.

Due to the challenges in obtaining microglia, induced pluripotent stem cells (iPSCs) could be valuable sources for acquiring microglia. Studies have shown that iPSCs can differentiate into microglia when exposed to specific cytokines (Speicher et al., 2019). The commercial space company SpaceX has reported that iPSCs isolated from AD and primary progressive multiple sclerosis (MS) patients can be sent into space and differentiated into microglia for investigating the relationship between microglia and neurodegenerative diseases in microgravity conditions (NASA.gov, 2019). These findings will contribute to the advancement of research on microglial activation in the future.

Microfluidic chip stimulations may emerge as suitable platforms for future *in vitro* spaceflight experiments, owing to their compact size and ease of automation. Furthermore, microfluidic chips can facilitate the co-culturing of cells, including vascular endothelial cells, glial cells, and microglia, to simulate substance exchange between the BBB and the brain within a microgravity environment. Real-time monitoring of cellular states can also be achieved by incorporating a monitoring module onto the microfluidic chip, as demonstrated in aerospace experiments involving macrophages (Shi et al., 2021). With advancements in research technology and the aerospace industry, further studies should be conducted on the changes in microglia in spaceflight conditions to provide strategies for ensuring the cognitive function and neural health of astronauts.

Furthermore, it should be noted that this paper does not address the potential damage to optic nerve function caused by microglia activation in microgravity, since the main focus is on the effects of microglia activation on cognitive and memory function in microgravity and spaceflight. However, approximately 40 to 60% of astronauts involved in long-term ISS space missions have reported the occurrence of spaceflight-associated neuro-ocular syndrome, with the phenomenon of ophthalmological findings and increased intracranial pressure (Rosenberg et al., 2021). Spaceflight-induced cephalad shift of body fluids was considered to be the inducement, possibly leading to elevated intracranial pressure (ICP) and intraocular pressure (IOP) (Taibbi et al., 2013). An increase in IOP is a major risk factor for glaucoma and triggers the responses of microglia and astroglia to participate in the process of neuroinflammation (Soto and Howell, 2014). Therefore, the symptoms of increased IOP experienced by astronauts during spaceflight may cause the activation of retinal microglia. Currently, only one study has reported IOP changes, optic nerve damage, and retinal microglial activation in rats after prolonged HU treatment (Li et al., 2022). However, further research on microglial activation in the eye associated with microgravity-related increases in IOP is needed.

## Author contributions

ZiL: Funding acquisition, Supervision, Writing – original draft. JW: Investigation, Methodology, Writing – original draft. TZ: Investigation, Writing – review & editing. YW: Investigation, Writing – review & editing. YX: Investigation, Writing – review & editing. ZoL: Supervision, Writing – review & editing. XL: Supervision, Writing – review & editing. XC: Writing – review & editing.

## References

[B1] Acharya M. M. Green K. N. Allen B. D. Najafi A. R. Syage A. Minasyan H. (2016). Elimination of microglia improves cognitive function following cranial irradiation. *Sci. Rep.* 6:31545. 10.1038/srep31545 27516055 PMC4981848

[B2] Adapala R. K. Thoppil R. J. Ghosh K. Cappelli H. C. Dudley A. C. Paruchuri S. (2016). Activation of mechanosensitive ion channel TRPV4 normalizes tumor vasculature and improves cancer therapy. *Oncogene* 35 314–322. 25867067 10.1038/onc.2015.83 PMC4948740

[B3] Alhadlaq M. W. Masocha W. (2023). Microglia and p38 MAPK inhibitors suppress development of mechanical allodynia in both sexes in a mouse model of antiretroviral-induced neuropathic pain. *Int. J. Mol. Sci.* 24:12805. 10.3390/ijms241612805 37628987 PMC10454318

[B4] Alvariño R. Alonso E. Tabudravu J. N. Pérez-Fuentes N. Alfonso A. Botana L. M. (2021). Tavarua deoxyriboside a and jasplakinolide as potential neuroprotective agents: effects on cellular models of oxidative stress and neuroinflammation. *ACS Chem. Neurosci.* 12 150–162. 10.1021/acschemneuro.0c00626 33353294

[B5] Andreev-Andrievskiy A. Popova A. Boyle R. Alberts J. Shenkman B. Vinogradova O. (2014). Mice in Bion-M 1 space mission: training and selection. *PLoS One* 9:e104830. 10.1371/journal.pone.0104830 25133741 PMC4136787

[B6] Anken R. H. Hilbig R. (2004). A drop-tower experiment to determine the threshold of gravity for inducing motion sickness in fish. *Adv. Space Res.* 34 1592–1597. 15880897 10.1016/j.asr.2004.01.023

[B7] Aoki Y. Dai H. Furuta F. Akamatsu T. Oshima T. Takahashi N. (2023). LOX-1 mediates inflammatory activation of microglial cells through the p38-MAPK/NF-κB pathways under hypoxic-ischemic conditions. *Cell Commun. Signal.* 21:126. 10.1186/s12964-023-01048-w PMC10236821 37268943

[B8] Aubert A. E. Larina I. Momken I. Blanc S. White O. Kim Prisk G. (2016). Towards human exploration of space: the THESEUS review series on cardiovascular, respiratory, and renal research priorities. *NPJ Microgr.* 2:16031. 10.1038/npjmgrav.2016.31 28725739 PMC5515532

[B9] Baba S. Smith T. Hellmann J. Bhatnagar A. Carter K. Vanhoover A. (2020). Space flight diet-induced deficiency and response to gravity-free resistive exercise. *Nutrients* 12:2400. 10.3390/nu12082400 32796546 PMC7468946

[B10] Bellone J. A. Gifford P. S. Nishiyama N. C. Hartman R. E. Mao X. W. (2016). Long-term effects of simulated microgravity and/or chronic exposure to low-dose gamma radiation on behavior and blood-brain barrier integrity. *NPJ Microgravity* 2:16019. 10.1038/npjmgrav.2016.19 PMC5516431 28725731

[B11] Blaber A. P. Zuj K. A. Goswami N. (2013). Cerebrovascular autoregulation: lessons learned from spaceflight research. *Eur. J. Appl. Physiol.* 113 1909–1917. 10.1007/s00421-012-2539-x 23132388

[B12] Bohlson S. S. Tenner A. J. (2023). Complement in the brain: contributions to neuroprotection, neuronal plasticity, and neuroinflammation. *Annu. Rev. Immunol.* 41 431–452. 10.1146/annurev-immunol-101921-035639 36750318

[B13] Borst A. Van Loon J. J. (2009). Technology and developments for the random positioning machine. *RPM. Microgr. Sci. Technol.* 21 287–292.

[B14] Borst K. Dumas A. A. Prinz M. (2021). Microglia: immune and non-immune functions. *Immunity* 54 2194–2208. 34644556 10.1016/j.immuni.2021.09.014

[B15] Brás J. P. Bravo J. Freitas J. Barbosa M. A. Santos S. G. Summavielle T. (2020). TNF-alpha-induced microglia activation requires miR-342: impact on NF-kB signaling and neurotoxicity. *Cell Death Dis.* 11:415. 10.1038/s41419-020-2626-6 32488063 PMC7265562

[B16] Brites D. Vaz A. R. (2014). Microglia centered pathogenesis in ALS: insights in cell interconnectivity. *Front. Cell Neurosci.* 8:117. 10.3389/fncel.2014.00117 24904276 PMC4033073

[B17] Brown G. C. Vilalta A. (2015). How microglia kill neurons. *Brain Res.* 1628 288–297. 26341532 10.1016/j.brainres.2015.08.031

[B18] Buchheim J. I. Matzel S. Rykova M. Vassilieva G. Ponomarev S. Nichiporuk I. (2019). Stress related shift toward inflammaging in cosmonauts after long-duration space flight. *Front. Physiol.* 10:85. 10.3389/fphys.2019.00085 30873038 PMC6401618

[B19] Buravkova L. B. Rudimov E. G. Andreeva E. R. Grigoriev A. I. (2018). The ICAM-1 expression level determines the susceptibility of human endothelial cells to simulated microgravity. *J. Cell Biochem.* 119 2875–2885. 10.1002/jcb.26465 29080356

[B20] Cassady K. Koppelmans V. Reuter-Lorenz P. De Dios Y. Gadd N. Wood S. (2016). Effects of a spaceflight analog environment on brain connectivity and behavior. *Neuroimage* 141 18–30. 27423254 10.1016/j.neuroimage.2016.07.029

[B21] Cekanaviciute E. Rosi S. Costes S. V. (2018). Central nervous system responses to simulated galactic cosmic rays. *Int. J. Mol. Sci.* 19:3669. 10.3390/ijms19113669 PMC6275046 30463349

[B22] Chang T. T. Spurlock S. M. Candelario T. L. Grenon S. M. Hughes-Fulford M. (2015). Spaceflight impairs antigen-specific tolerance induction in vivo and increases inflammatory cytokines. *FASEB J.* 29 4122–4132. 10.1096/fj.15-275073 26085131 PMC6137544

[B23] Chelyshev Y. A. Muhamedshina Y. O. Povysheva T. V. Shaymardanova G. F. Rizvanov A. A. Nigmetzyanova M. V. (2014). Characterization of spinal cord glial cells in a model of hindlimb unloading in mice. *Neuroscience* 280 328–339. 10.1016/j.neuroscience.2014.09.004 25218808

[B24] Chen W. F. Shih Y. H. Liu H. C. Cheng C. I. Chang C. I. Chen C. Y. (2022). 6-methoxyflavone suppresses neuroinflammation in lipopolysaccharide- stimulated microglia through the inhibition of TLR4/MyD88/p38 MAPK/NF-κB dependent pathways and the activation of HO-1/NQO-1 signaling. *Phytomedicine* 99:154025. 10.1016/j.phymed.2022.154025 35272244

[B25] Chen X. Wanggou S. Bodalia A. Zhu M. Dong W. Fan J. J. (2018). A feedforward mechanism mediated by mechanosensitive ion channel PIEZO1 and tissue mechanics promotes glioma aggression. *Neuron* 100 799–815.e7. 10.1016/j.neuron.2018.09.046 30344046

[B26] Cogoli-Greuter M. (2014). The lymphocyte story–an overview of selected highlights on the in vitro activation of human lymphocytes in space. *Microgr. Sci. Technol.* 25 343–352.

[B27] Colonna M. Butovsky O. (2017). Microglia function in the central nervous system during health and neurodegeneration. *Annu. Rev. Immunol.* 35 441–468. 28226226 10.1146/annurev-immunol-051116-052358 PMC8167938

[B28] Crucian B. Babiak-Vazquez A. Johnston S. Pierson D. L. Ott C. M. Sams C. (2016). Incidence of clinical symptoms during long-duration orbital spaceflight. *Int. J. Gen. Med.* 9 383–391. 27843335 10.2147/IJGM.S114188 PMC5098747

[B29] Crucian B. Stowe R. Mehta S. Uchakin P. Quiriarte H. Pierson D. (2013). Immune system dysregulation occurs during short duration spaceflight on board the space shuttle. *J. Clin. Immunol.* 33 456–465. 10.1007/s10875-012-9824-7 23100144

[B30] de Araújo Boleti A. P. de Oliveira Flores T. M. Moreno S. E. Anjos L. D. Mortari M. R. Migliolo L. (2020). Neuroinflammation: an overview of neurodegenerative and metabolic diseases and of biotechnological studies. *Neurochem. Int.* 136:104714. 10.1016/j.neuint.2020.104714 32165170

[B31] De la Torre G. G. (2014). Cognitive neuroscience in space. *Life* 4 281–294. 25370373 10.3390/life4030281 PMC4206847

[B32] Desai R. I. Limoli C. L. Stark C. E. L. Stark S. M. (2022). Impact of spaceflight stressors on behavior and cognition: a molecular, neurochemical, and neurobiological perspective. *Neurosci. Biobehav. Rev.* 138:104676. 10.1016/j.neubiorev.2022.104676 35461987

[B33] Dhapola R. Hota S. S. Sarma P. Bhattacharyya A. Medhi B. Reddy D. H. (2021). Recent advances in molecular pathways and therapeutic implications targeting neuroinflammation for Alzheimer’s disease. *Inflammopharmacology* 29 1669–1681. 34813026 10.1007/s10787-021-00889-6 PMC8608577

[B34] Du Preez A. Onorato D. Eiben I. Musaelyan K. Egeland M. Zunszain P. (2021). Chronic stress followed by social isolation promotes depressive-like behaviour, alters microglial and astrocyte biology and reduces hippocampal neurogenesis in male mice. *Brain Behav. Immun.* 91 24–47. 10.1016/j.bbi.2020.07.015 32755644

[B35] ElGindi M. Sapudom J. Ibrahim I. H. Al-Sayegh M. Chen W. Garcia-Sabate A. (2021). May the force be with you (or not): the immune system under microgravity. *Cells* 10:1941. 10.3390/cells10081941 34440709 PMC8391211

[B36] European Space Agency (2022). *Drop Towers.* Available online at: https://www.esa.int/Education/ESA_Academy_Experiments_programme/Drop_Towers (accessed August 12, 2023).

[B37] Feng X. Frias E. S. Paladini M. S. Chen D. Boosalis Z. Becker M. (2021). Functional role of brain-engrafted macrophages against brain injuries. *J. Neuroinflammation* 18:232. 10.1186/s12974-021-02290-0 PMC8520231 34654458

[B38] Ferranti F. Del Bianco M. Pacelli C. (2020). Advantages and limitations of current microgravity platforms for space biology research. *Appl. Sci.* 11:68.

[B39] Frigeri A. Iacobas D. A. Iacobas S. Nicchia G. P. Desaphy J. F. Camerino D. C. (2008). Effect of microgravity on gene expression in mouse brain. *Exp. Brain Res.* 191 289–300. 18704384 10.1007/s00221-008-1523-5 PMC2651838

[B40] Gallardo-Fernández M. Hornedo-Ortega R. Alonso-Bellido I. M. Rodríguez-Gómez J. A. Troncoso A. M. García-Parrilla M. C. (2019). Hydroxytyrosol decreases LPS- and α-synuclein-induced microglial activation in vitro. *Antioxidants* 9:36. 10.3390/antiox9010036 31906130 PMC7022576

[B41] Gemignani A. Piarulli A. Menicucci D. Laurino M. Rota G. Mastorci F. (2014). How stressful are 105 days of isolation? sleep EEG patterns and tonic cortisol in healthy volunteers simulating manned flight to mars. *Int. J. Psychophysiol.* 93 211–219. 10.1016/j.ijpsycho.2014.04.008 24793641

[B42] Ginhoux F. Greter M. Leboeuf M. Nandi S. See P. Gokhan S. (2010). Fate mapping analysis reveals that adult microglia derive from primitive macrophages. *Science* 330 841–845. 10.1126/science.1194637 20966214 PMC3719181

[B43] Gitik M. Reichert F. Rotshenker S. (2010). Cytoskeleton plays a dual role of activation and inhibition in myelin and zymosan phagocytosis by microglia. *FASEB J.* 24 2211–2221. 10.1096/fj.09-146118 20179145

[B44] Goldman D. H. Dykstra T. Smirnov I. Blackburn S. M. Da Mesquita S. Kipnis J. (2022). Age-associated suppression of exploratory activity during sickness is linked to meningeal lymphatic dysfunction and microglia activation. *Nat. Aging* 2 704–713. 10.1038/s43587-022-00268-y 37065770 PMC10103743

[B45] Grimm D. Wehland M. Pietsch J. Aleshcheva G. Wise P. van Loon J. (2014). Growing tissues in real and simulated microgravity: new methods for tissue engineering. *Tissue Eng. Part B Rev.* 20 555–566. 10.1089/ten.TEB.2013.0704 24597549 PMC4241976

[B46] Gulen M. F. Samson N. Keller A. Schwabenland M. Liu C. Glück S. (2023). cGAS-STING drives ageing-related inflammation and neurodegeneration. *Nature* 620 374–380. 10.1038/s41586-023-06373-1 37532932 PMC10412454

[B47] Guo S. Wang H. Yin Y. (2022). Microglia polarization from M1 to M2 in neurodegenerative diseases. *Front. Aging Neurosci.* 14:815347. 10.3389/fnagi.2022.815347 35250543 PMC8888930

[B48] Han X. Cheng X. Xu J. Liu Y. Zhou J. Jiang L. (2022). Activation of TREM2 attenuates neuroinflammation via PI3K/Akt signaling pathway to improve postoperative cognitive dysfunction in mice. *Neuropharmacology* 219:109231. 10.1016/j.neuropharm.2022.109231 36041498

[B49] Hansen D. V. Hanson J. E. Sheng M. (2017). Microglia in Alzheimer’s disease. *J. Cell Biol.* 217 459–472. 29196460 10.1083/jcb.201709069 PMC5800817

[B50] Hay J. R. Johnson V. E. Young A. M. Smith D. H. Stewart W. (2015). Blood-brain barrier disruption is an early event that may persist for many years after traumatic brain injury in humans. *J. Neuropathol. Exp. Neurol.* 74 1147–1157. 26574669 10.1097/NEN.0000000000000261 PMC8744142

[B51] He D. Fu S. Ye B. Wang H. He Y. Li Z. (2023). Activation of HCA2 regulates microglial responses to alleviate neurodegeneration in LPS-induced in vivo and in vitro models. *J. Neuroinflammation* 20:86. 10.1186/s12974-023-02762-5 36991440 PMC10053461

[B52] Hefendehl J. K. Neher J. J. Sühs R. B. Kohsaka S. Skodras A. Jucker M. (2014). Homeostatic and injury-induced microglia behavior in the aging brain. *Aging Cell* 13 60–69. 10.1111/acel.12149 23953759 PMC4326865

[B53] Heneka M. T. Golenbock D. T. Latz E. (2015). Innate immunity in Alzheimer’s disease. *Nat. Immunol.* 16 229–236. 25689443 10.1038/ni.3102

[B54] Holley J. M. Stanbouly S. Pecaut M. J. Willey J. S. Delp M. Mao X. W. (2022). Characterization of gene expression profiles in the mouse brain after 35 days of spaceflight mission. *NPJ Microgr.* 8:35. 10.1038/s41526-022-00217-4 35948598 PMC9365836

[B55] Hong S. Beja-Glasser V. F. Nfonoyim B. M. Frouin A. Li S. Ramakrishnan S. (2016). Complement and microglia mediate early synapse loss in Alzheimer mouse models. *Science* 352 712–716. 27033548 10.1126/science.aad8373 PMC5094372

[B56] Hooper C. Taylor D. L. Pocock J. M. (2005). Pure albumin is a potent trigger of calcium signalling and proliferation in microglia but not macrophages or astrocytes. *J. Neurochem.* 92 1363–1376. 15748155 10.1111/j.1471-4159.2005.02982.x

[B57] Jassam Y. N. Izzy S. Whalen M. McGavern D. B. El Khoury J. (2017). Neuroimmunology of traumatic brain injury: time for a paradigm shift. *Neuron* 95 1246–1265. 10.1016/j.neuron.2017.07.010 28910616 PMC5678753

[B58] Kawabori M. Yenari M. A. (2015). The role of the microglia in acute CNS injury. *Metab. Brain Dis.* 30 381–392. 24682762 10.1007/s11011-014-9531-6 PMC4180000

[B59] Kessler R. C. (1997). The effects of stressful life events on depression. *Annu. Rev. Psychol.* 48 191–214. 9046559 10.1146/annurev.psych.48.1.191

[B60] Kim R. E. Shin C. Y. Han S. H. Kwon K. J. (2020). Astaxanthin suppresses PM2.5-induced neuroinflammation by regulating Akt Phosphorylation in BV-2 microglial cells. *Int. J. Mol. Sci.* 21:7227. 10.3390/ijms21197227 33008094 PMC7582569

[B61] Kim Y. H. Koh H. K. Kim D. S. (2010). Down-regulation of IL-6 production by astaxanthin via ERK-, MSK-, and NF-κB-mediated signals in activated microglia. *Int. Immunopharmacol.* 10 1560–1572. 20932499 10.1016/j.intimp.2010.09.007

[B62] Kodaira S. Naito M. Uchihori Y. Hashimoto H. Yano H. Yamagishi A. (2021). Space radiation dosimetry at the exposure facility of the international space station for the tanpopo mission. *Astrobiology* 21 1473–1478. 34348047 10.1089/ast.2020.2427

[B63] Krieger S. S. Zwart S. R. Mehta S. Wu H. Simpson R. J. Smith S. M. (2021). Alterations in saliva and plasma cytokine concentrations during long-duration spaceflight. *Front. Immunol.* 12:725748. 10.3389/fimmu.2021.725748 34504500 PMC8422944

[B64] Krukowski K. Feng X. Paladini M. S. Chou A. Sacramento K. Grue K. (2018a). Temporary microglia-depletion after cosmic radiation modifies phagocytic activity and prevents cognitive deficits. *Sci. Rep.* 8: 7857. 29777152 10.1038/s41598-018-26039-7 PMC5959907

[B65] Krukowski K. Grue K. Frias E. S. Pietrykowski J. Jones T. Nelson G. (2018b). Female mice are protected from space radiation-induced maladaptive responses. *Brain Behav. Immun.* 74 106–120. 10.1016/j.bbi.2018.08.008 30107198 PMC8715721

[B66] Kumar A. Loane D. J. (2012). Neuroinflammation after traumatic brain injury: opportunities for therapeutic intervention. *Brain Behav. Immun.* 26 1191–1201. 22728326 10.1016/j.bbi.2012.06.008

[B67] Li Q. Zhao H. F. Zhang Z. F. Liu Z. G. Pei X. R. Wang J. B. (2009). Long-term administration of green tea catechins prevents age-related spatial learning and memory decline in C57BL/6 J mice by regulating hippocampal cyclic amp-response element binding protein signaling cascade. *Neuroscience* 159 1208–1215. 10.1016/j.neuroscience.2009.02.008 19409206

[B68] Li S. Song Q. Wu B. Kan G. Wang F. Yang J. (2022). Structural damage to the rat eye following long-term simulated weightlessness. *Exp. Eye Res.* 223:109200. 10.1016/j.exer.2022.109200 35932903

[B69] Liao Y. Zhang J. Huang Z. Xi Y. Zhang Q. Zhu T. (2012). Altered baseline brain activity with 72 h of simulated microgravity–initial evidence from resting-state fMRI. *PLoS One* 7:e52558. 10.1371/journal.pone.0052558 23285086 PMC3528642

[B70] Lim S. L. Tran D. N. Zumkehr J. Chen C. Ghiaar S. Kieu Z. (2018). Inhibition of hematopoietic cell kinase dysregulates microglial function and accelerates early stage Alzheimer’s disease-like neuropathology. *Glia* 66 2700–2718. 10.1002/glia.23522 30277607 PMC6645690

[B71] Lin T. Du J. Liu L. Wu Z. Kong X. Liu Y. (2020). Treatment with minocycline suppresses microglia activation and reverses neural stem cells loss after simulated microgravity. *Biomed. Res. Int.* 2020:7348745. 10.1155/2020/7348745 32382569 PMC7196960

[B72] Liu H. J. Cai Q. Ji G. Y. Zhan Z. Jiang J. D. Zhu M. C. (2000). [Changes of mRNA expression of IL-1beta and TNF-alpha in rat brains after repeated exposures to +Gz]. *Space Med. Med. Eng.* 13 371–373. 11894876

[B73] Lü D. Sun S. Zhang F. Luo C. Zheng L. Wu Y. (2019). Microgravity-induced hepatogenic differentiation of rBMSCs on board the SJ-10 satellite. *FASEB J.* 33 4273–4286. 10.1096/fj.201802075R 30521385

[B74] Manzey D. Lorenz B. (1998). Mental performance during short-term and long-term spaceflight. *Brain Res. Brain Res. Rev.* 28 215–221. 9795225 10.1016/s0165-0173(98)00041-1

[B75] Mao X. W. Nishiyama N. C. Byrum S. D. Stanbouly S. Jones T. Holley J. (2020). Spaceflight induces oxidative damage to blood-brain barrier integrity in a mouse model. *FASEB J.* 34 15516–15530. 10.1096/fj.202001754R 32981077 PMC8191453

[B76] Mao X. W. Nishiyama N. C. Pecaut M. J. Campbell-Beachler M. Gifford P. Haynes K. E. (2016). Simulated microgravity and low-dose/low-dose-rate radiation induces oxidative damage in the mouse brain. *Radiat. Res.* 185 647–657. 27243749 10.1667/RR14267.1

[B77] Mao X. W. Sandberg L. B. Gridley D. S. Herrmann E. C. Zhang G. Raghavan R. (2018). Proteomic analysis of mouse brain subjected to spaceflight. *Int. J. Mol. Sci.* 20:7. 10.3390/ijms20010007 PMC6337482 30577490

[B78] Mhatre S. D. Iyer J. Petereit J. Dolling-Boreham R. M. Tyryshkina A. Paul A. M. (2022). Artificial gravity partially protects space-induced neurological deficits in Drosophila melanogaster. *Cell Rep.* 40:111279. 10.1016/j.celrep.2022.111279 36070701 PMC10503492

[B79] Miyanishi K. Sato A. Kihara N. Utsunomiya R. Tanaka J. (2021). Synaptic elimination by microglia and disturbed higher brain functions. *Neurochem. Int.* 142:104901. 10.1016/j.neuint.2020.104901 33181238

[B80] Morey-Holton E. R. Globus R. K. (2002). Hindlimb unloading rodent model: technical aspects. *J. Appl. Physiol.* 92 1367–1377. 11895999 10.1152/japplphysiol.00969.2001

[B81] NASA.gov (2019). *The Effects of Microgravity on Microglia 3-Dimensional Models of Parkinson’s Disease and Multiple Sclerosis.* Available online at: https://www.nasa.gov/mission/station/research-explorer/investigation/?#id=7976 (accessed August 14, 2023).

[B82] Nassef M. Z. Kopp S. Melnik D. Corydon T. J. Sahana J. Krüger M. (2019). Short-term microgravity influences cell adhesion in human breast cancer cells. *Int. J. Mol. Sci.* 20:5730. 10.3390/ijms20225730 PMC6887954 31731625

[B83] Nelson G. A. (2016). Space radiation and human exposures. a primer. *Radiat. Res.* 185 349–358. 27018778 10.1667/RR14311.1

[B84] No author list (1992). COSMOS 2044 mission. *J. Appl. Physiol.* 73 1S–200S. 1526933

[B85] Ohira T. Kawano F. Goto K. Kaji H. Ohira Y. (2022). Responses of neuromuscular properties to unloading and potential countermeasures during space exploration missions. *Neurosci. Biobehav. Rev.* 136:104617. 10.1016/j.neubiorev.2022.104617 35283170

[B86] Ojo B. Rezaie P. Gabbott P. L. Davies H. Colyer F. Cowley T. R. (2012). Age-related changes in the hippocampus (loss of synaptophysin and glial-synaptic interaction) are modified by systemic treatment with an NCAM-derived peptide. FGL. *Brain Behav. Immun.* 26 778–788. 10.1016/j.bbi.2011.09.013 21986303

[B87] Pani G. Samari N. Quintens R. de Saint-Georges L. Meloni M. Baatout S. (2013). Morphological and physiological changes in mature in vitro neuronal networks towards exposure to short-, middle- or long-term simulated microgravity. *PLoS One* 8:e73857. 10.1371/journal.pone.0073857 24066080 PMC3774774

[B88] Pani G. Verslegers M. Quintens R. Samari N. de Saint-Georges L. van Oostveldt P. (2016). Combined exposure to simulated microgravity and acute or chronic radiation reduces neuronal network integrity and survival. *PLoS One* 11:e0155260. 10.1371/journal.pone.0155260 27203085 PMC4874625

[B89] Paolicelli R. C. Sierra A. Stevens B. Tremblay M. E. Aguzzi A. Ajami B. (2022). Microglia states and nomenclature: a field at its crossroads. *Neuron* 110 3458–3483. 36327895 10.1016/j.neuron.2022.10.020 PMC9999291

[B90] Parhizkar S. Gent G. Chen Y. Rensing N. Gratuze M. Strout G. (2023). Sleep deprivation exacerbates microglial reactivity and Aβ deposition in a TREM2-dependent manner in mice. *Sci. Transl. Med.* 15:eade6285. 10.1126/scitranslmed.ade6285 PMC10449561 37099634

[B91] Parihar V. K. Allen B. D. Caressi C. Kwok S. Chu E. Tran K. K. (2016). Cosmic radiation exposure and persistent cognitive dysfunction. *Sci. Rep.* 6:34774. 10.1038/srep34774 PMC5056393 27721383

[B92] Parihar V. K. Angulo M. C. Allen B. D. Syage A. Usmani M. T. Passerat de la Chapelle E. (2020). Sex-Specific cognitive deficits following space radiation exposure. *Front. Behav. Neurosci.* 14:535885. 10.3389/fnbeh.2020.535885 33192361 PMC7525092

[B93] Parihar V. K. Maroso M. Syage A. Allen B. D. Angulo M. C. Soltesz I. (2018). Persistent nature of alterations in cognition and neuronal circuit excitability after exposure to simulated cosmic radiation in mice. *Exp. Neurol.* 305 44–55. 10.1016/j.expneurol.2018.03.009 29540322

[B94] Paulsen K. Tauber S. Dumrese C. Bradacs G. Simmet D. M. Gölz N. (2015). Regulation of ICAM-1 in cells of the monocyte/macrophage system in microgravity. *Biomed. Res. Int.* 2015:538786. 10.1155/2015/538786 25654110 PMC4309248

[B95] Petit G. Cebolla A. M. Fattinger S. Petieau M. Summerer L. Cheron G. (2019). Local sleep-like events during wakefulness and their relationship to decreased alertness in astronauts on ISS. *NPJ Microgr.* 5:10. 10.1038/s41526-019-0069-0 31069253 PMC6497715

[B96] Pluvinage J. V. Haney M. S. Smith B. A. H. Sun J. Iram T. Bonanno L. (2019). CD22 blockade restores homeostatic microglial phagocytosis in ageing brains. *Nature* 568 187–192. 30944478 10.1038/s41586-019-1088-4 PMC6574119

[B97] Porseva V. V. Shilkin V. V. Strelkov A. A. Krasnov I. B. Masliukov P. M. (2017). Changes in the neurochemical composition of motor neurons of the spinal cord in mice under conditions of space flight. *Bull. Exp. Biol. Med.* 162 336–339. 10.1007/s10517-017-3609-1 28091925

[B98] Raber J. Allen A. R. Sharma S. Allen B. Rosi S. Olsen R. H. (2016). Effects of proton and combined proton and (56)fe radiation on the hippocampus. *Radiat. Res.* 185 20–30. 10.1667/RR14222.1 26720797

[B99] Rahimifard M. Maqbool F. Moeini-Nodeh S. Niaz K. Abdollahi M. Braidy N. (2017). Targeting the TLR4 signaling pathway by polyphenols: a novel therapeutic strategy for neuroinflammation. *Ageing Res. Rev.* 36 11–19. 10.1016/j.arr.2017.02.004 28235660

[B100] Ratushnyy A. Yakubets D. Andreeva E. Buravkova L. (2019). Simulated microgravity modulates the mesenchymal stromal cell response to inflammatory stimulation. *Sci. Rep.* 9:9279. 10.1038/s41598-019-45741-8 31243304 PMC6594925

[B101] Rienecker K. D. A. Paladini M. S. Grue K. Krukowski K. Rosi S. (2021). Microglia: ally and enemy in deep space. *Neurosci. Biobehav. Rev.* 126 509–514. 10.1016/j.neubiorev.2021.03.036 33862064

[B102] Risch N. Herrell R. Lehner T. Liang K. Y. Eaves L. Hoh J. (2009). Interaction between the serotonin transporter gene (5-HTTLPR), stressful life events, and risk of depression: a meta-analysis. *JAMA* 301 2462–2471. 19531786 10.1001/jama.2009.878 PMC2938776

[B103] Rojo A. I. McBean G. Cindric M. Egea J. Lopez M. G. Rada P. (2014). Redox control of microglial function: molecular mechanisms and functional significance. *Antioxid. Redox Signal.* 21 1766–1801. 24597893 10.1089/ars.2013.5745 PMC4186766

[B104] Rola R. Fishman K. Baure J. Rosi S. Lamborn K. R. Obenaus A. (2008). Hippocampal neurogenesis and neuroinflammation after cranial irradiation with (56)Fe particles. *Radiat. Res.* 169 626–632. 10.1667/RR1263.1 18494546 PMC2583781

[B105] Roll J. P. Popov K. Gurfinkel V. Lipshits M. Andre-Deshays C. Gilhodes J. C. (1993). Sensorimotor and perceptual function of muscle proprioception in microgravity. *J. Vestib. Res.* 3 259–273. 8275261

[B106] Ronca A. E. Moyer E. L. Talyansky Y. Lowe M. Padmanabhan S. Choi S. (2019). Behavior of mice aboard the international space station. *Sci. Rep.* 9: 4717. 30976012 10.1038/s41598-019-40789-y PMC6459880

[B107] Rosenberg M. J. Coker M. A. Taylor J. A. Yazdani M. Matheus M. G. Blouin C. K. (2021). Comparison of dural venous sinus volumes before and after flight in astronauts with and without spaceflight-associated neuro-ocular syndrome. *JAMA Netw. Open* 4:e2131465. 10.1001/jamanetworkopen.2021.31465 34705011 PMC8552058

[B108] Rosito M. Sanchini C. Gosti G. Moreno M. De Panfilis S. Giubettini M. (2023). Microglia reactivity entails microtubule remodeling from acentrosomal to centrosomal arrays. *Cell Rep.* 42:112104. 10.1016/j.celrep.2023.112104 36787220 PMC10423306

[B109] Rubinstein L. Kiffer F. Puukila S. Lowe M. G. Goo B. Luthens A. (2022). Mitochondria-targeted human catalase in the mouse longevity MCAT model mitigates head-tilt bedrest-induced neuro-inflammation in the hippocampus. *Life* 12:1838. 10.3390/life12111838 36362993 PMC9695374

[B110] Sajdel-Sulkowska E. M. Nguon K. Sulkowski Z. L. Lipinski B. (2007). Potential role of oxidative stress in mediating the effect of altered gravity on the developing rat cerebellum. *Adv. Space Res.* 40 1414–1420. 10.1016/j.asr.2007.08.004 18438448 PMC2344128

[B111] Sandal G. M. (2000). Coping in Antarctica: is it possible to generalize results across settings? *Aviat. Space Environ. Med.* 71 A37–A43. 10993307

[B112] Sandal G. M. Vaernes R. Ursin H. (1995). Interpersonal relations during simulated space missions. *Aviat. Space Environ. Med.* 66 617–624. 7575308

[B113] Santucci D. Kawano F. Ohira T. Terada M. Nakai N. Francia N. (2012). Evaluation of gene, protein and neurotrophin expression in the brain of mice exposed to space environment for 91 days. *PLoS One* 7:e40112. 10.1371/journal.pone.0040112 22808101 PMC3392276

[B114] Schneider S. Brummer V. Carnahan H. Dubrowski A. Askew C. D. Struder H. K. (2008). What happens to the brain in weightlessness? a first approach by EEG tomography. *Neuroimage* 42 1316–1323. 10.1016/j.neuroimage.2008.06.010 18606233

[B115] Segarra M. Aburto M. R. Acker-Palmer A. (2021). Blood-brain barrier dynamics to maintain brain homeostasis. *Trends Neurosci.* 44 393–405. 33423792 10.1016/j.tins.2020.12.002

[B116] Shen Z. Xu H. Song W. Hu C. Guo M. Li J. (2021). Galectin-1 ameliorates perioperative neurocognitive disorders in aged mice. *CNS Neurosci. Ther.* 27 842–856. 10.1111/cns.13645 33942523 PMC8193703

[B117] Shi L. Li B. Chen G. Huang Y. Tian Z. Zhang L. (2022). MEF2D participates in microglia-mediated neuroprotection in cerebral ischemia-reperfusion rats. *Shock* 57 118–130. 10.1097/SHK.0000000000001844 34905532

[B118] Shi L. Tian H. Wang P. Li L. Zhang Z. Zhang J. (2021). Spaceflight and simulated microgravity suppresses macrophage development via altered RAS/ERK/NFκB and metabolic pathways. *Cell. Mol. Immunol.* 18 1489–1502. 31900461 10.1038/s41423-019-0346-6 PMC8167113

[B119] Simpson D. S. A. Oliver P. L. (2020). ROS generation in microglia: understanding oxidative stress and inflammation in neurodegenerative disease. *Antioxidants* 9:743. 10.3390/antiox9080743 PMC7463655 32823544

[B120] Smith S. M. Abrams S. A. Davis-Street J. E. Heer M. O’Brien K. O. Wastney M. E. (2014). Fifty years of human space travel: implications for bone and calcium research. *Annu. Rev. Nutr.* 34 377–400. 10.1146/annurev-nutr-071813-105440 24995691

[B121] Solis A. G. Bielecki P. Steach H. R. Sharma L. Harman C. C. D. Yun S. (2019). Mechanosensation of cyclical force by PIEZO1 is essential for innate immunity. *Nature* 573 69–74. 31435009 10.1038/s41586-019-1485-8 PMC6939392

[B122] Song S. Wang S. Pigott V. M. Jiang T. Foley L. M. Mishra A. (2018). Selective role of Na(+) /H(+) exchanger in Cx3cr1(+) microglial activation, white matter demyelination, and post-stroke function recovery. *Glia* 66 2279–2298. 10.1002/glia.23456 30043461 PMC6430713

[B123] Song S. Yu L. Hasan M. N. Paruchuri S. S. Mullett S. J. Sullivan M. L. (2022). Elevated microglial oxidative phosphorylation and phagocytosis stimulate post-stroke brain remodeling and cognitive function recovery in mice. *Commun. Biol.* 5:35. 10.1038/s42003-021-02984-4 35017668 PMC8752825

[B124] Soto I. Howell G. R. (2014). The complex role of neuroinflammation in glaucoma. *Cold Spring Harb. Perspect. Med.* 4:a017269. 10.1101/cshperspect.a017269 PMC4109578 24993677

[B125] Speicher A. M. Wiendl H. Meuth S. G. Pawlowski M. (2019). Generating microglia from human pluripotent stem cells: novel in vitro models for the study of neurodegeneration. *Mol. Neurodegener.* 14:46. 10.1186/s13024-019-0347-z 31856864 PMC6921408

[B126] Stence N. Waite M. Dailey M. E. (2001). Dynamics of microglial activation: a confocal time-lapse analysis in hippocampal slices. *Glia* 33 256–266. 11241743

[B127] Stevens B. Allen N. J. Vazquez L. E. Howell G. R. Christopherson K. S. Nouri N. (2007). The classical complement cascade mediates CNS synapse elimination. *Cell* 131 1164–1178. 18083105 10.1016/j.cell.2007.10.036

[B128] Su W. Aloi M. S. Garden G. A. (2016). MicroRNAs mediating CNS inflammation: small regulators with powerful potential. *Brain Behav. Immun.* 52 1–8. 10.1016/j.bbi.2015.07.003 26148445 PMC5030842

[B129] Subhramanyam C. S. Wang C. Hu Q. Dheen S. T. (2019). Microglia-mediated neuroinflammation in neurodegenerative diseases. *Semin. Cell Dev. Biol.* 94 112–120. 31077796 10.1016/j.semcdb.2019.05.004

[B130] Sun E. Motolani A. Campos L. Lu T. (2022). The pivotal role of NF-kB in the pathogenesis and therapeutics of Alzheimer’s disease. *Int. J. Mol. Sci.* 23:8972. 10.3390/ijms23168972 PMC9408758 36012242

[B131] Tahimic C. G. T. Paul A. M. Schreurs A. S. Torres S. M. Rubinstein L. Steczina S. (2019). Influence of social isolation during prolonged simulated weightlessness by hindlimb unloading. *Front. Physiol.* 10:1147. 10.3389/fphys.2019.01147 31572207 PMC6753329

[B132] Taibbi G. Cromwell R. L. Kapoor K. G. Godley B. F. Vizzeri G. (2013). The effect of microgravity on ocular structures and visual function: a review. *Surv. Ophthalmol.* 58 155–163. 23369516 10.1016/j.survophthal.2012.04.002

[B133] Takata F. Nakagawa S. Matsumoto J. Dohgu S. (2021). Blood-brain barrier dysfunction amplifies the development of neuroinflammation: understanding of cellular events in brain microvascular endothelial cells for prevention and treatment of BBB dysfunction. *Front. Cell Neurosci.* 15:661838. 10.3389/fncel.2021.661838 34588955 PMC8475767

[B134] Thiel C. S. Tauber S. Lauber B. Polzer J. Seebacher C. Uhl R. (2019). Rapid morphological and cytoskeletal response to microgravity in human primary macrophages. *Int. J. Mol. Sci.* 20:2402. 10.3390/ijms20102402 31096581 PMC6567851

[B135] Van Baarsen B. Ferlazzo F. Ferravante D. DiNocera F. Jörgensen J. Smit J. H. (2009). “Digging into space psychology and isolation: the Mars520 LODGEAD study. preliminary results of the Mars105 pilot study,” in *Proceedings of the 60th International Astronautical Congress*, (Daejeon).

[B136] Victor M. B. Leary N. Luna X. Meharena H. S. Scannail A. N. Bozzelli P. L. (2022). Lipid accumulation induced by APOE4 impairs microglial surveillance of neuronal-network activity. *Cell Stem. Cell* 29 1197–1212.e8. 10.1016/j.stem.2022.07.005 35931030 PMC9623845

[B137] Vu A. P. Lam D. Denney C. Lee K. V. Plemel J. R. Jackson J. (2023). Social isolation produces a sex- and brain region-specific alteration of microglia state. *Eur. J. Neurosci.* 57 1481–1497. 10.1111/ejn.15966 36918398

[B138] Wang T. Chen H. Lv K. Ji G. Zhang Y. Wang Y. (2017). iTRAQ-based proteomics analysis of hippocampus in spatial memory deficiency rats induced by simulated microgravity. *J. Proteomics* 160 64–73. 10.1016/j.jprot.2017.03.013 28341594

[B139] Wang T. Ruan B. Wang J. Zhou Z. Zhang X. Zhang C. (2022). Activation of NLRP3-Caspase-1 pathway contributes to age-related impairments in cognitive function and synaptic plasticity. *Neurochem. Int.* 152:105220. 10.1016/j.neuint.2021.105220 34743016

[B140] Wang Z. Zhou L. An D. Xu W. Wu C. Sha S. (2019). TRPV4-induced inflammatory response is involved in neuronal death in pilocarpine model of temporal lobe epilepsy in mice. *Cell Death Dis.* 10:386. 10.1038/s41419-019-1612-3 PMC6522539 31097691

[B141] Wolf S. A. Boddeke H. W. Kettenmann H. (2017). Microglia in physiology and disease. *Annu. Rev. Physiol.* 79 619–643. 27959620 10.1146/annurev-physiol-022516-034406

[B142] Wu X. T. Yang X. Tian R. Li Y. H. Wang C. Y. Fan Y. B. (2022). Cells respond to space microgravity through cytoskeleton reorganization. *FASEB J.* 36:e22114. 10.1096/fj.202101140R 35076958

[B143] Wu Y. Wu H. Zeng J. Pluimer B. Dong S. Xie X. (2021). Mild traumatic brain injury induces microvascular injury and accelerates Alzheimer-like pathogenesis in mice. *Acta Neuropathol. Commun.* 9:74. 10.1186/s40478-021-01178-7 33892818 PMC8063402

[B144] Wuest S. L. Gantenbein B. Ille F. Egli M. (2018). Electrophysiological experiments in microgravity: lessons learned and future challenges. *NPJ Microgravity* 4:7. 10.1038/s41526-018-0042-3 29619409 PMC5876337

[B145] Wuest S. L. Stern P. Casartelli E. Egli M. (2017). Fluid dynamics appearing during simulated microgravity using random positioning machines. *PLoS One* 12:e0170826. 10.1371/journal.pone.0170826 28135286 PMC5279744

[B146] Xavier A. L. Menezes J. R. Goldman S. A. Nedergaard M. (2014). Fine-tuning the central nervous system: microglial modelling of cells and synapses. *Philos. Trans. R. Soc. Lond. B Biol. Sci.* 369:20130593. 10.1098/rstb.2013.0593 25225087 PMC4173279

[B147] Xia Q. Mao M. Zhan G. Luo Z. Zhao Y. Li X. (2023). SENP3-mediated deSUMOylation of c-Jun facilitates microglia-induced neuroinflammation after cerebral ischemia and reperfusion injury. *iScience* 26:106953. 10.1016/j.isci.2023.106953 37332598 PMC10272502

[B148] Xie N. Xiao C. Shu Q. Cheng B. Wang Z. Xue R. (2023). Cell response to mechanical microenvironment cues via Rho signaling: from mechanobiology to mechanomedicine. *Acta Biomater.* 159 1–20. 10.1016/j.actbio.2023.01.039 36717048

[B149] Xue R. Wan Y. Sun X. Zhang X. Gao W. Wu W. (2019). Nicotinic mitigation of neuroinflammation and oxidative stress after chronic sleep deprivation. *Front. Immunol.* 10:2546. 10.3389/fimmu.2019.02546 31736967 PMC6828928

[B150] Yan R. Liu H. Lv F. Deng Y. Li Y. (2021). Rac1/Wave2/Arp3 pathway mediates rat blood-brain barrier dysfunction under simulated microgravity based on proteomics strategy. *Int. J. Mol. Sci.* 22:5165. 10.3390/ijms22105165 34068233 PMC8153163

[B151] Yang X. Xu S. Qian Y. Xiao Q. (2017). Resveratrol regulates microglia M1/M2 polarization via PGC-1α in conditions of neuroinflammatory injury. *Brain Behav. Immun.* 64 162–172. 28268115 10.1016/j.bbi.2017.03.003

[B152] Ye L. Shu S. Jia J. Sun M. Xu S. Bao X. (2023). Absent in melanoma 2 mediates aging-related cognitive dysfunction by acting on complement-dependent microglial phagocytosis. *Aging Cell* 22:e13860. 10.1111/acel.13860 37177836 PMC10352562

[B153] Zhang L. Dong L. Y. Li Y. J. Hong Z. Wei W. S. (2012). miR-21 represses FasL in microglia and protects against microglia-mediated neuronal cell death following hypoxia/ischemia. *Glia* 60 1888–1895. 10.1002/glia.22404 22907769

[B154] Zhang L. Zhang J. Jiang X. Yang L. Zhang Q. Wang B. (2020). Hydroxytyrosol Inhibits LPS-induced neuroinflammatory responses via suppression of TLR-4-mediated NF-κB P65 activation and ERK signaling pathway. *Neuroscience* 426 189–200. 31866556 10.1016/j.neuroscience.2019.12.005

[B155] Zhang X. Surguladze N. Slagle-Webb B. Cozzi A. Connor J. R. (2006). Cellular iron status influences the functional relationship between microglia and oligodendrocytes. *Glia* 54 795–804. 16958088 10.1002/glia.20416

